# Lifesaving surgery for a ruptured invasive thymoma using the hemi-clamshell approach: a case report

**DOI:** 10.1186/s40792-019-0594-9

**Published:** 2019-02-19

**Authors:** Nobuyuki Yoshiyasu, Fumitsugu Kojima, Yuya Ishikawa, Toru Bando

**Affiliations:** Department of Thoracic Surgery, St. Luke’s International Hospital, 9-1 Akashi-cho, Chuo-ku, Tokyo, 104-8560 Japan

**Keywords:** Emergency surgery, Hemi-clamshell approach, Ruptured invasive thymoma

## Abstract

**Background:**

Among anterior mediastinal tumors, a teratoma is known to rupture with growth, but there have been few previous reports about thymoma rupture. We here report a rare case of an invasive thymoma with intrapulmonary and intrathoracic rupture requiring emergency life-saving surgery. To our knowledge, this is the first such case in the literature.

**Case presentation:**

A 56-year-old woman suddenly experienced right precordial pain and hemoptysis. Enhanced computed tomography revealed a large mediastinal tumor pressing against the pulmonary hilar vascularity, with extravasation of blood into the right lung. Tumor rupture into the lungs was suspected. Given the deterioration of her respiratory status and hemodynamics, thymomectomy with removal of the involved tissues was urgently performed using the hemi-clamshell approach and intrapericardial dissection, with veno-arterial extracorporeal membrane oxygenation on standby. She survived, and no recurrence has been noted for 2 years postoperatively.

**Conclusions:**

A large thymoma can suddenly rupture into the thorax, similar to the rupture of a teratoma. Additionally, in cases with hemoptysis, an appropriate procedure should be selected to reach both the pulmonary hilum and thorax for complete resection, as hemoptysis might suggest tumor invasion into the lungs.

## Background

Among anterior mediastinal tumors, a teratoma is known to rupture with growth, but a ruptured thymoma, requiring emergency surgery, has rarely been reported, and the use of the hemi-clamshell approach in such a case has not been described to date. However, this approach does have some advantages for patients with hemodynamic instability due to compression by a large tumor or its severe adhesion to the pulmonary hilum [1].

Here, we present a rare case of an invasive thymoma with intrapulmonary and intrathoracic rupture treated with emergency thymomectomy involving the hemi-clamshell approach and intrapericardial dissection. To our knowledge, this is the first such case in the literature.

## Case presentation

**Fig. 1:**
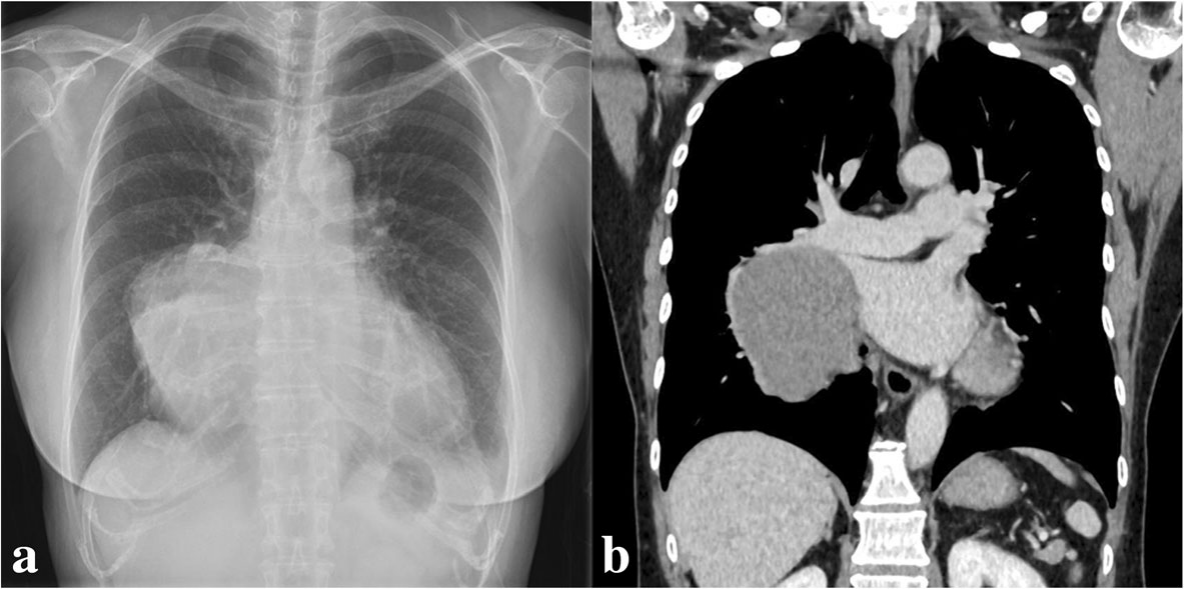
Chest radiography (**a**) and chest computed tomography (**b**) at the first visit show a large mass in the right lower lung field

**Fig. 2:**
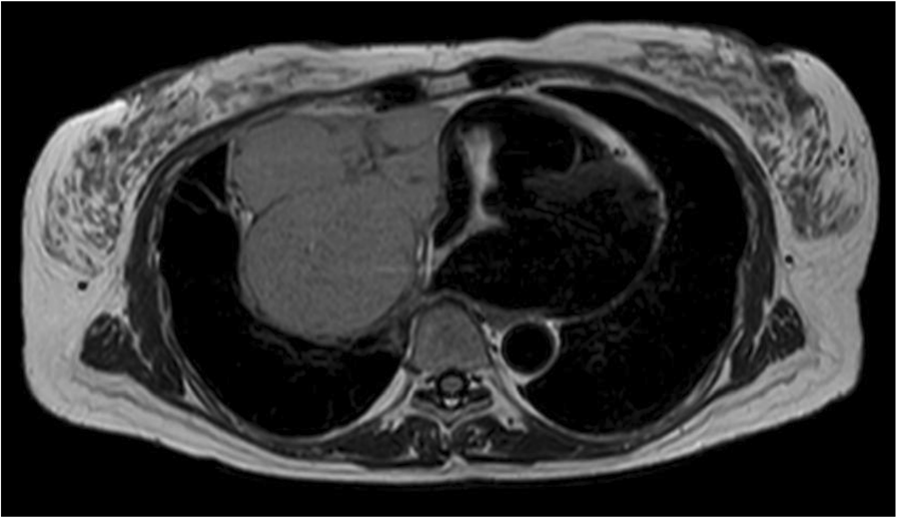
Magnetic resonance imaging (T2-weighted imaging) shows a capsulated mass (10 × 11 cm in diameter) at the right anterior mediastinum

A mass shadow was incidentally detected in the right lower lung field on routine chest radiography and chest computed tomography (Fig. 1a, b) in a 56-year-old woman with a menopausal disorder. Magnetic resonance imaging (T2-weighted imaging) revealed a capsulated mass (10 × 11 cm in diameter) extending from the anterior mediastinum toward the middle mediastinum (Fig. 2). On fluorine-18 fluorodeoxyglucose (FDG) positron-emission tomography, increased FDG uptake was seen, with a maximum standardized uptake value of 9.6. Surgery was planned for the mediastinal tumor suspected to be a low-grade thymoma. As her menopausal disorder was being treated with estrogen therapy, which is associated with an increased risk of venous thromboembolism and pulmonary thromboembolism, we scheduled surgery 1 month after therapy discontinuation.

**Fig. 3:**
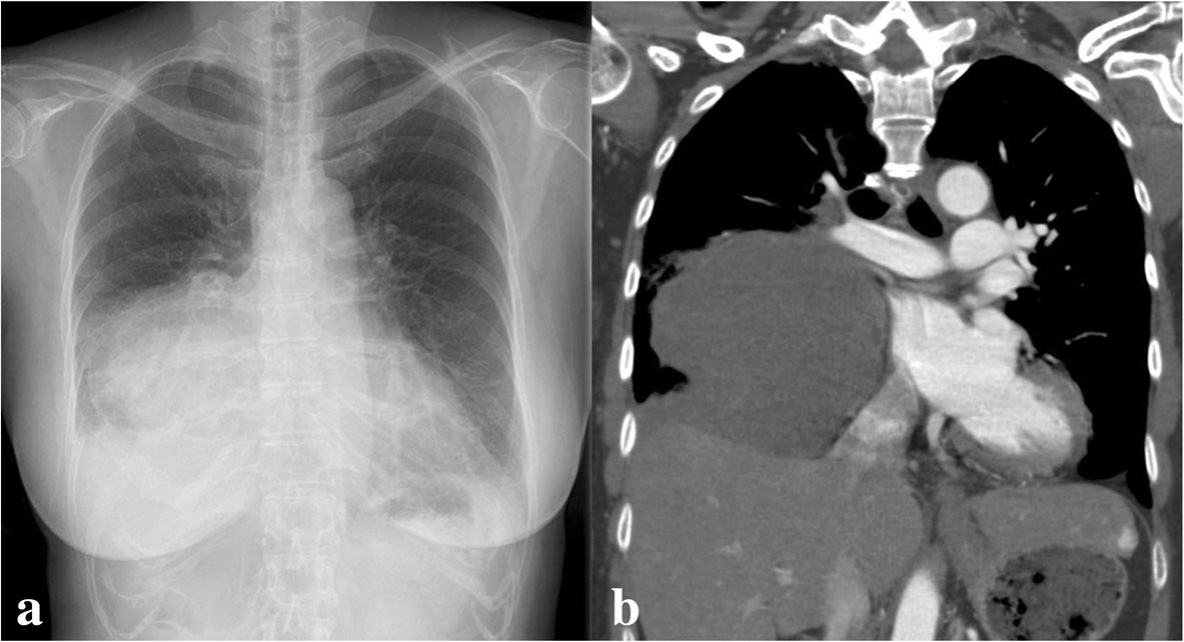
Second chest radiography (**a**) shows an enlarged mass with an irregular shape. Enhanced computed tomography (**b**) after the admission demonstrates occlusion of the right inferior pulmonary vein due to tumor compression

However, before the scheduled date, she suddenly complained of acute pain in the right precordial region, which radiated toward the back. The pain exacerbated and hemoptysis occurred when she coughed; thus, she visited the emergency room at our hospital. Her vital signs were stable, except for tachypnea with oxygen desaturation. Auscultation indicated decreased breath sounds in the right lower lung. Her electrocardiogram was normal, without ST elevation. Second chest radiography showed an enlarged tumor with an irregular shape (Fig. 3a), whereas enhanced computed tomography revealed a diffuse ground-glass shadow in the right lung, with occlusion of the intermediate bronchus and right inferior pulmonary vein by the tumor (Fig. 3b). Furthermore, there was extravasation of blood into the right lung, as well as pleural effusion. We presumed that her sudden-onset symptoms were caused by intrapulmonary rupture of the mediastinal tumor.

**Fig. 4:**
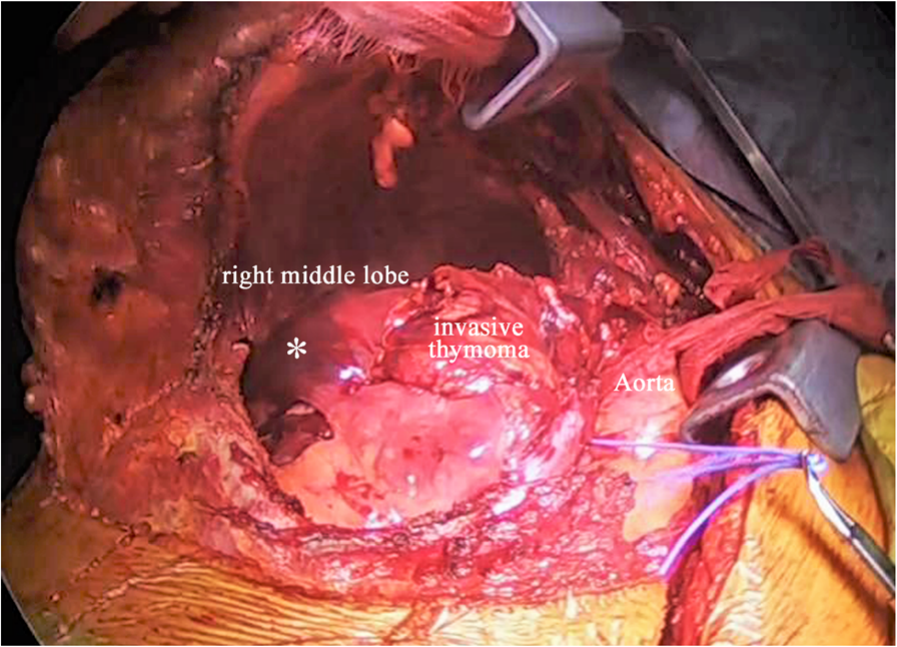
The surgical view in the hemi-clamshell approach with intrapericardial dissection. The right main pulmonary artery was tied using blue rubber bands, while the ascending aorta was covered with gauze. The right lung with the thymoma appeared dark red, indicating a hematoma (asterisk)

Emergency surgery was performed with veno-arterial extracorporeal membrane oxygenation (VA-ECMO) on standby, because of the possibility of deterioration of the respiratory status and hemodynamics during anesthesia induction. After anesthesia induction on one-lung ventilation, we confirmed that blood pressure and oxygen saturation did not change markedly owing to alteration of body position. We initially performed median sternotomy and found that the large tumor had infiltrated strongly into the right lung and phrenic nerve on the pericardium around the hilar area. Thus, after securing the right main pulmonary artery with an incision into the serous pericardium between the superior vena cava (SVC) and ascending aorta, we conducted a hemi-clamshell approach to obtain a better surgical view (Fig. 4). Finally, we performed thymomectomy with right pneumonectomy and partial resection of the pericardium and completely excised the thymoma, without requiring VA-ECMO. The right phrenic nerve had to be sacrificed, and a part of the diaphragm was resected with a stapler because of suspected direct invasion. She had an uneventful postoperative recovery, and she was discharged from the hospital 9 days after the surgery.

**Fig. 5:**
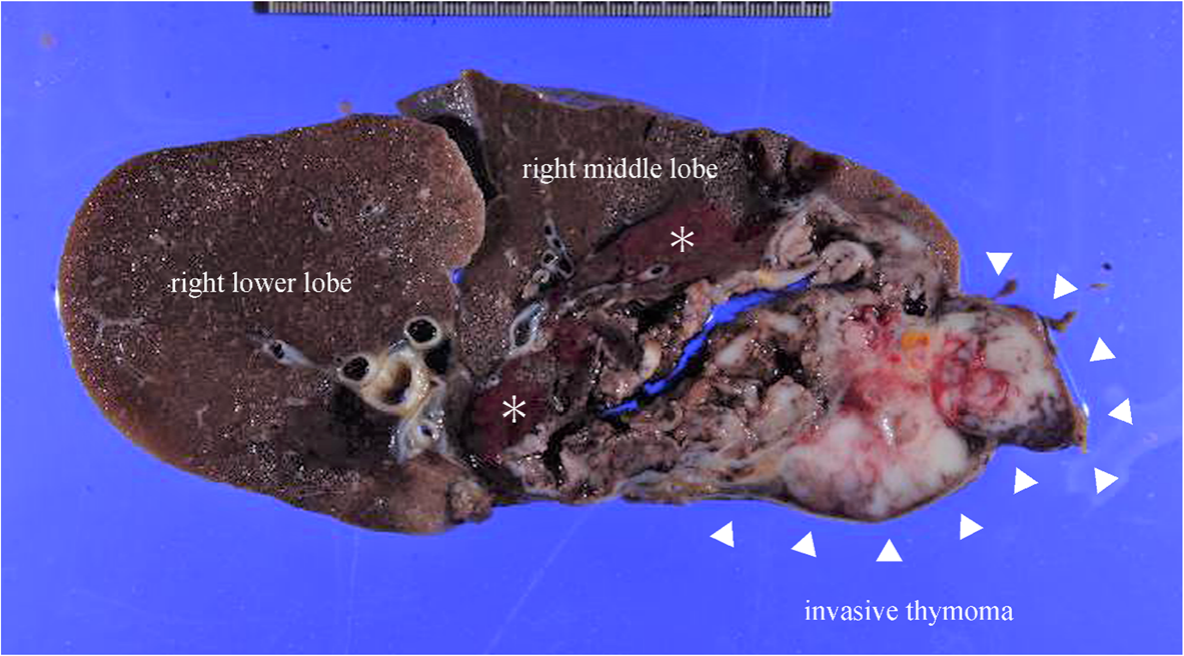
A histopathological specimen shows strong infiltration of the invasive thymoma into the right middle lobe. The white solid medullary tumor demonstrates bleeding (asterisk) into the lung

Pathological examination revealed that the mediastinal tumor was stage III according to the Masaoka–Koga classification and a type B2 invasive thymoma according to the World Health Organization histological classification. The tumor had infiltrated into the right upper and middle lobes and the pericardium. Vascular breakdown due to tumor compression resulted in pulmonary hemorrhage (Fig. 5). Although the margin was negative, radiation therapy was performed. She survived, and no recurrence has been detected for 2 years postoperatively.

## Discussion

An invasive thymoma occurs in the anterior mediastinum and frequently infiltrates into the mediastinal pleura, lungs, pericardium, or blood vessels. The tumor may progress in a variety of ways. Cases involving extension into the SVC and right atrium or thymic veins have been reported previously [2, 3]. In the present case, the invasive thymoma mainly developed at the side of the thoracic cavity. The direction of progression may depend on the origin of the tumor, even in the same anterior mediastinum.

Among anterior mediastinal tumors, mediastinal teratomas are known to infiltrate into the thorax, as these tumors contain some pancreatic tissue, which secretes pancreatic enzymes and causes histolysis and consequent rupture [4]. In contrast, an invasive thymoma with intrathoracic rupture has rarely been reported [5–10], but has been suggested to be caused by ischemia of the tumor wall owing to rapid growth. To our knowledge, this is the first case of intrapulmonary and intrathoracic rupture of an invasive thymoma requiring emergency surgery. In this case, marked pulmonary hemorrhage was observed in the middle lobe specimen (Fig. 4). Microscopic observation indicated widespread collapse of blood vessels and lung tissues. We assumed that the internal pressure of the tumor had increased as it was sandwiched between the intermediate bronchus and the heart, and it then ruptured into the lungs, where the pressure was most easily released owing to tumor invasion of the lungs. Six cases of penetration of thymomas into the thorax have been reported to date [5–10]. These patients complained of chest pain and/or dyspnea, but none of them had hemoptysis, as in the present case (Table 1). When a patient complains of not only chest discomfort, but also hemoptysis, it should be suspected that the thymoma has infiltrated into the lungs and has progressed to a relatively advanced stage. Therefore, an appropriate approach should be selected to facilitate access to the pulmonary hilum for complete resection in order to remove the tissues surrounding the lung.

**Table 1:** Ruptured thymoma case series

First author [Reference]	Year	Age/sex	Specimen size (cm)	Symptoms	Histology (WHO classification)	Masaoka–Koga classification	Surgical approach
Caplin [5]	1985	51/M	―	Chest pain and dyspnea	―	―	Posterolateral thoracotomy
Templeton [6]	1988	63/M	―	Chest pain and dyspnea with hypotension	Malignant thymoma	III	Biopsy only
Fukuse [7]	1991	70/M	―*	Dyspnea	―	―	Median sternotomy with anterolateral thoracotomy
Shimokawa [8]	2001	71/F	6 × 4.5 × 3.5	Chest pain and dyspnea with mild hypotension	AB	II	Upper-part sternotomy with hemi-transection of the sternum to the fourth intercostal space
Santoprete [9]	2007	73/F	12.5 × 14.5 × 12	Chest pain	AB	II	Clamshell
Hokka [10]	2015	77/F	7.2 × 4.3 × 4.3	Chest pain	B1	II	Anterolateral thoracotomy with video-assisted thoracoscopic surgery
Present case	2018	56/F	10.5 × 11 × 4.6	Chest pain with hemoptysis	B2	III	Hemi-clamshell with sternotomy

―No description

*The authors reported the diameter of the thymoma to be 0.7 cm, but the maximum diameter of the cystic tumor was not specified

In the present case, we successfully performed complete resection of the tumor by using the hemi-clamshell approach and performing intrapericardial dissection in pneumonectomy. When an intrathoracic operation is required for a mediastinal tumor, the hemi-clamshell approach provides a better operating field than median sternotomy [11]. The hemi-clamshell approach can provide multi-angle views of the pulmonary hilum, posterior–anterior mediastinum, and diaphragm. Thus, this approach for lobectomy or even pneumonectomy with large thymomectomy will be useful to control hilar and interlobar anatomic dissection. Furthermore, if the tumor adheres severely to the surrounding tissues at the pulmonary hilum, intrapericardial dissection should be performed to control unexpected hemorrhage. After incision of the fibrous and serous pericardium via median sternotomy, the right main pulmonary artery can be secured on the dorsal side between the SVC and ascending aorta.

In conclusion, a large invasive thymoma can suddenly rupture into the thorax, similar to the rupture of a teratoma. Additionally, in cases with hemoptysis, an appropriate procedure, such as the hemi-clamshell approach, should be used to reach both the pulmonary hilum and thorax for complete resection, as hemoptysis might suggest tumor invasion into the lungs.

## Abbreviations

FDG: Fluorine-18 fluorodeoxyglucose
SVC: Superior vena cava
VA-ECMO: Veno-arterial extracorporeal membrane oxygenation
